# Molecular epidemiology and antimicrobial resistance of *Salmonella* at the human–macaque–environment interface in Thailand: A One Health surveillance study

**DOI:** 10.14202/vetworld.2025.1549-1560

**Published:** 2025-06-15

**Authors:** Suchawan Pornsukarom, Daraka Tongthainan, Phairot Phromwat, Suwarak Wannaratana, Kulchai Nakbubpa, Sarut Muangsri

**Affiliations:** 1 Faculty of Veterinary Medicine, Rajamangala University of Technology Tawan-ok, Si Racha, Chonburi, Thailand; 2 Department of National Parks, Wildlife and Plant Conservation, Bangkok, Thailand; 3 Veterinary Diagnostic Center, Rajamangala University of Technology Tawan-ok, Si Racha, Chonburi, Thailand

**Keywords:** antimicrobial resistance, environmental reservoirs, macaques, molecular typing, One Health, *Salmonella*, Thailand, zoonosis

## Abstract

**Background and Aim:**

The close interaction between humans and free-ranging macaques in urbanized environments raises concerns about the potential transmission of antimicrobial-resistant zoonotic pathogens. This study applied a One Health approach to estimate the prevalence, serovar distribution, antimicrobial resistance (AMR), and genetic diversity of *Salmonella* spp. in long-tailed macaques (*Macaca fascicularis*) and environmental sources in Chonburi, Thailand.

**Materials and Methods:**

A total of 313 samples – including 224 rectal swabs from macaques and 89 environmental samples (pooled macaque feces, stray dog feces, soil, feed, and drain water) – were collected from Si Racha and Sattahip districts between April and July 2023. *Salmonella* isolation was conducted using conventional culture methods, followed by confirmation through serotyping and polymerase chain reaction targeting the *inv*A gene. Antimicrobial susceptibility testing was performed against 14 agents using broth microdilution. Multi-locus sequence typing and *16S rRNA* gene sequencing were conducted to assess phylogenetic diversity.

**Results:**

The overall prevalence of *Salmonella* was 2.88%, with all positive samples detected in the Si Racha district. Environmental samples had a significantly higher prevalence (8.89%) than macaque rectal swabs (0.45%; odds ratio = 22; 95% confidence interval: 2.71–178.84; p = 0.0002). Six distinct serovars were identified, with *Salmonella* Corvallis predominating in macaque feces. Among the nine isolates, 77.78% exhibited resistance, primarily to tetracycline and ampicillin. Notably, 85.71% of AMR strains from environmental samples were multidrug-resistant (MDR), showing resistance to ≥6 antimicrobials. Phylogenetic analysis revealed genetic heterogeneity, with no clear clustering by source or serovar.

**Conclusion:**

This study underscores the circulation of MDR *Salmonella* within macaques and their surrounding environments, implicating environmental reservoirs in potential zoonotic and reverse zoonotic transmission. The findings advocate for public awareness initiatives, environmental hygiene improvements, and integrative One Health strategies to mitigate AMR dissemination at the human–animal–ecosystem interface.

## INTRODUCTION

**Figure 1:**
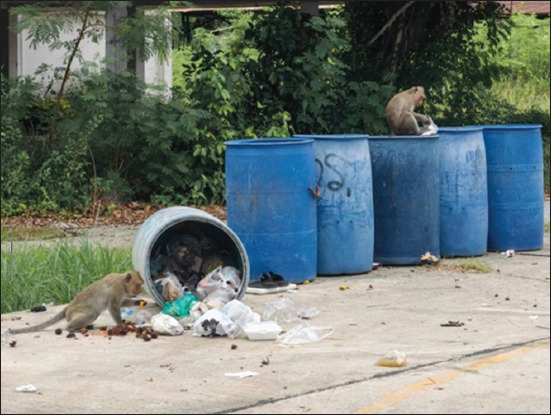
Free-ranging long-tailed macaques in the community, Bangphra, Si Racha, Chonburi, Thailand.

Macaques (*Macaca* spp.) are among the most widely distributed non-human primates, frequently inhabiting human-modified environments, including urban and peri-urban settings across South and Southeast Asia [1–5]. Species such as the rhesus macaque (*Macaca mulatta*), toque macaque (*Macaca sinica*), and long-tailed macaque (*Macaca fascicularis*) have become increasingly synanthropic due to rapid land-use changes and expanding urbanization, leading to local overpopulation and heightened human–wildlife conflicts [2, 6]. In many urban contexts, macaques have adapted to anthropogenic food sources, such as household refuse, food waste, and sewage, often accessing them by foraging in trash bins, scavenging, or snatching food directly from humans (Figure 1). These primates also frequent tourist sites, temples, and archeological areas, further intensifying their interactions with people and increasing the risk of zoonotic disease transmission.

Among the zoonotic threats, antimicrobial-resistant *Salmonella* spp. represent a critical public health concern. Rahman *et al*. [3], Boonkusol *et al*. [4], and Tegner *et al*. [5] have documented the presence of antimicrobial-resistant *Salmonella* and other zoonotic bacteria in non-human primates, including *Campylobacter* spp. and *Staphylococcus* spp., particularly in contexts of close human–animal interaction. In Thailand, urban and wild populations of long-tailed macaques have been shown to harbor AMR Enterobacteriaceae, such as *Salmonella* spp., Shiga toxin-producing *Escherichia coli* (STEC), and *Shigella* spp., further implicating these animals in the environmental persistence and transmission of antimicrobial-resistant pathogens [4, 7]. Given that macaques are seldom treated with antimicrobials, the emergence of resistance within their microbiota suggests indirect acquisition from contaminated environmental reservoirs or reverse zoonotic transmission. Within the One Health framework, such ecosystems – shared by humans, domestic animals, and wildlife – serve as convergence points for the exchange and dissemination of resistant bacteria [8, 9].

Chonburi province in eastern Thailand, a hub within the Eastern Economic Corridor (EEC), exemplifies this dynamic interface. The districts of Si Racha and Sattahip are characterized by mixed-use landscapes combining urban development, natural habitats, and tourist infrastructure. These areas have experienced a rise in free-ranging macaque populations, which are attracted to human settlements, exacerbating public health concerns and necessitating integrated surveillance efforts.

Despite the increasing recognition of free-ranging macaques as potential reservoirs of zoonotic pathogens, there remains a paucity of molecular epidemiological data on *Salmonella* spp., particularly AMR strains, at the human–macaque interface in Thailand. Existing studies have largely focused on non-invasive fecal sampling and lack comprehensive integration of environmental surveillance or genetic characterization of isolates. Furthermore, the role of shared environments as intermediate reservoirs for AMR determinants, especially in rapidly urbanizing and touristic regions such as Chonburi has not been systematically investigated. The transmission dynamics between macaques, humans, and environmental sources, including potential reverse zoonotic pathways, thus remain poorly understood.

This study aimed to estimate the prevalence, AMR profiles, and genetic diversity of *Salmonella* spp. isolated from free-ranging long-tailed macaques *(M. fascicularis*) and their surrounding environments in Chonburi, Thailand. By employing culture-based isolation, antimicrobial susceptibility testing (AST), serotyping, multi-locus sequence typing (MLST), and 16S rRNA gene-based phylogenetic analysis, this research seeks to elucidate the potential role of environmental reservoirs in the transmission of antimicrobial-resistant *Salmonella* within a One Health framework. The findings are intended to inform risk assessment and support the development of integrated surveillance and control strategies in regions where human–wildlife interfaces are intensifying.

## MATERIALS AND METHODS

### Ethical approval

Ethical approval for the study was granted by the Animal Ethics Committee of Rajamangala University of Technology, Tawan-ok, Chonburi, Thailand (Permit No.: RMUTTO-ACUC-2-2023-010).

### Study period and location

**Figure 2:**
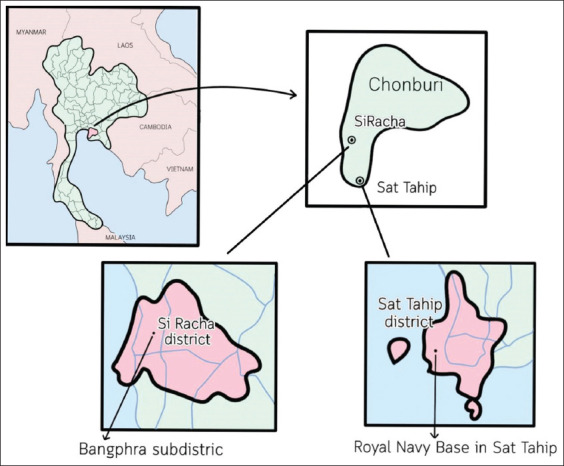
The map of the study sites in Sriracha and Sattahip districts, Chonburi, Thailand [Source: The map was generated using Procreate 5.3.4].

A cross-sectional study was conducted from April to July 2023 to estimate the prevalence of *Salmonella* and AMR profiles in macaques and environmental samples from the Si Racha and Sattahip districts of Chonburi Province, Thailand. These locations are geographically situated at coordinates 13.1425° N, 101.0485° E (Si Racha) and 12.6945° N, 100.9129° E (Sattahip) (Figure 2). The study was conducted within the framework of a macaque population control program implemented by the Bang Phra Subdistrict Administrative Organization (SAO) in Si Racha and the Khet Udomsak Municipality in Sattahip.

Macaques were captured using baited cages containing seasonal fruits, grains, and corn. These cages were strategically positioned across Bang Phra Subdistrict in Si Racha and around the Military Camp, Krom Luang Chumphon Court in Sattahip Naval Base. Captured macaques were subsequently transported to temporary veterinary stations at Wat Phrommawat in Si Racha and the Navy Camp in Sattahip, where sterilization procedures and sample collection were performed.

### Sample size calculation

The sample size for each site was determined proportionally to the local macaque density based on surveillance and pre-visit programs conducted by SAO officers and regional veterinarians. Sample size estimation was performed using the single-population proportion formula, with an expected prevalence of 5% based on a previous study by Rahman *et al*. [3], a 5% margin of error, and a 95% confidence interval (CI), calculated using Epitools Epidemiological Calculators [10]. However, the final sample size at each site depended on the availability and physical condition of macaques within the cages. In total, 224 monkeys (Si Racha = 127; Sattahip = 97) were used for sample collection. In addition, 89 environmental samples were collected, comprising 73 pooled fecal samples and 16 environmental specimens. The sample distribution is summarized in Table 1.

**Table 1:** Prevalence of *Salmonella* isolates recovered from macaque and environmental sites in Chonburi, Thailand.

Type of sample	Si Racha (n = 183)		Sattahip (n = 130)		Total (n = 313)	
		
	No. of samples	*Salmonella* (%)	No. of samples	*Salmonella* (%)	No. of samples	*Salmonella* (%)
Macaque						
Rectal swab (n = 224)	127	1 (0.79)	97	0	224	1 (0.45)
Environment (n = 89)	56	8 (14.29)	33	0	89	8 (8.99)
Pooled macaque feces (n = 70)	40	5 (12.5)	30	0	70	5 (7.14)
Pooled dog feces (n = 3)	3	1 (33.33)	0	0	3	1 (33.33)
Soil (n = 10)	10	1 (10)	0	0	10	1 (10)
Feed (n = 4)	3	1 (33.33)	1	0	4	1 (25)
Drain water (n = 2)	0	0	2	0	2	0
Total (n = 313)	183	9 (4.92)	130	0	313	9 (2.88)

### Sample collection

#### Macaque rectal swabs

All 224 macaques underwent physical examination and were sedated using intramuscular administration of alfaxalone (6 mg/kg; Alfaxan, Jurox, MO, USA). Respiratory rate, heart rate, and body temperature were continuously monitored during the anesthetic procedure. Rectal swabs were collected at multiple time points, depending on the availability of macaques at the sterilization station. Sterile swabs were inserted 3 cm past the anal verge and gently rotated 720° within the rectum. Each swab was transferred into a labeled 2-mL cryotube containing phosphate-buffered saline, stored in a 4°C icebox, and subsequently preserved at −80°C within 4 h before further processing. During postoperative monitoring, macaques were held in cages until they had fully recovered and then released at their original capture locations. All procedures were conducted by wildlife practitioners in accordance with the Guide for the Care and Use of Laboratory Animals published by the U.S. National Institutes of Health [11].

#### Environmental sampling

A total of 89 environmental samples (each at least 50 g or 50 mL) were collected from the areas surrounding Wat Phrommawat and the Navy Camp. These included 70 pooled monkey feces samples, 10 soil samples, 4 monkey feed samples, 3 pooled stray dog feces samples, and 2 drain water samples (Table 1). Pooled macaque feces and feed samples were randomly collected from floors and transport vehicles at multiple time points following the arrival of macaques at the temporary sterilization stations using sterile scoops and zip-lock plastic bags. Pooled dog feces and other environmental specimens were collected at a single time point per location. Drain water samples were obtained using 120-mL sterile containers from areas near the sterilization station at the Navy Camp. Soil samples (25 cm depth) and dog feces were collected from the surroundings of Wat Phrommawat and adjacent communities using sterile scoops and zip-lock plastic bags. All environmental samples were stored in a 4°C icebox and transferred to −80°C within 4 h for subsequent processing.

### *Salmonella* isolation and confirmation

All 313 samples (224 macaques and 89 environmental) were processed weekly upon arrival at the Faculty of Veterinary Medicine, Rajamangala University of Technology Tawan-ok, Chonburi, following previously described protocols for *Salmonella* isolation [12]. Briefly, each sample was homogenized with buffered peptone water (BPW; Difco, Becton Dickinson, MD, USA) and incubated at 37°C for 24 h. The pre-enriched suspension was transferred to Rappaport-Vassiliadis (RV) enrichment broth (HiMedia, Mumbai, India) and incubated at 42°C for 24 h. A loopful of enriched RV suspension was streaked onto xylose-lysine-deoxycholate (XLD) agar (Difco, Becton Dickinson, MD, USA) and incubated overnight at 37°C. A single black colony from the XLD agar was selected and streaked into triple sugar iron (TSI) slants (Becton Dickinson, NJ, USA) for biochemical confirmation. Presumptive *Salmonella* isolates positive on TSI were subjected to polymerase chain reaction (PCR) targeting the *inv*A gene [13], with all reactions performed in duplicate. The primer sequences and annealing temperatures are presented in Table 2. Reference *Salmonella* strains, *Salmonella* Kentucky (SK) [14] and *Salmonella* Weltevreden (SW) [15], obtained from the Veterinary Diagnostic Center, Rajamangala University of Technology Tawan-ok, were used as positive controls. No-template negative controls were included in each PCR batch for validation. Confirmed isolates were stored in Brucella broth (HiMedia, Mumbai, India) supplemented with 10% glycerol (Kemaus, NSW, Australia) at −80°C for further analysis.

**Table 2:** Oligonucleotide primers used for polymerase chain reaction amplification in this study.

Target gene	Primer	Sequence (5’ ⟶ 3’)	Annealing temperature (°C)	No. of cycles	Product size (bp)	References
*inv*A	*inv*Af	GTGAAATTATCGCCACGTTCGGGCAA	55	35	284	[13]
	*inv*Ar	TCATCGCACCGTCAAAGGAACC				
*aro*C	*aro*Cf	CCTGGCACCTCGCGCTATAC	55	34	826	[18, 20]
	*aro*Cr	CCACACACGGATCGTGGCG				
*dna*N	*dna*Nf	ATGAAATTTACCGTTGAACGTGA	55	34	833	[18, 19]
	*dna*Nr	AATTTCTCATTCGAGAGGATTGC				
*hem*D	*hem*Df	GAAGCGTTAGTGAGCCGTCTGCG	55	34	666	[18, 20]
	*hem*Dr	ATCAGCGACCTTAATATCTTGCCA				
*his*D	*his*Df	GAAACGTTCCATTCCGCGCAGAC	55	34	894	[18, 19]
	*his*Dr	CTGAACGGTCATCCGTTTCTG				
*pur*E	*pur*Ef	ATGTCTTCCCGCAATAATCC	55	34	510	[18, 19]
	*pur*Er	TCATAGCGTCCCCCGCGGATC				
*suc*A	*suc*Af	CGCGCTCAAACAGACCTAC	55	34	643	[18, 20]
	*suc*Ar	GACGTGGAAAATCGGCGCC				
*thr*A	*thr*Af	GTCACGGTGATCGATCCGGT	55	34	852	[18, 20]
	*thr*Ar	CACGATATTGATATTAGCCCG				
16s rRNA	16SF1	TGTTGTGGTTAATAACCGCA	55	35	572	[22]
	16SIII	CACAAATCCATCTCTGGA				

### *Salmonella* serotyping

All confirmed *Salmonella* isolates (n = 9) were cultured overnight at 37°C on tryptic soy agar (TSA; Difco, Becton Dickinson, MD, USA) and subjected to serotyping through slide agglutination using the Kauffman–White scheme and commercial antisera (S&A Reagents Lab, Bangkok, Thailand).

### AST

Minimum inhibitory concentrations (MICs) and AMR profiles were determined for all confirmed *Salmonella* isolates using the broth microdilution method. Bacterial suspensions were standardized to 0.5 McFarland before inoculation onto Sensititre® EUVSEC panels (Thermo Fisher Scientific, MA, USA). Testing was conducted in duplicate. The panel comprised 14 antimicrobials with respective ranges: ampicillin (AMP; 1–64 μg/mL), azithromycin (AZI; 2–64 μg/mL), chloramphenicol (CHL; 8–128 μg/mL), ciprofloxacin (CIP; 0.015–8 μg/mL), colistin (COL; 1–16 μg/mL), cefotaxime (FOT; 0.25–4 μg/mL), gentamicin (GEN; 0.5–32 μg/mL), meropenem (MEM; 0.03–16 μg/mL), nalidixic acid (NAL; 4–128 μg/mL), sulfamethoxazole (SMX; 8–1024 μg/mL), ceftazidime (TAZ; 0.5–8 μg/mL), tetracycline (TET; 2–64 μg/mL), tigecycline (TGC; 0.25–8 μg/mL), and trimethoprim (TMP; 0.25–32 μg/mL). MIC results were interpreted following guidelines from the Clinical and Laboratory Standards Institute (CLSI M100-ED33) [16] and the European Committee on Antimicrobial Susceptibility Testing [17]. *E. coli* ATCC 25922 was used as the quality control strain. Isolates showing resistance to three or more antimicrobial classes were classified as multidrug-resistant (MDR).

### MLST

Genomic DNA was extracted from 17 *Salmonella* isolates, including nine from this study (S09, S10, S11, S13, S14, S15, S16, S21, and S22), six from livestock, pork, and wildlife sources (S12, S20, S23, S24, S25, and S26), and two reference strains (SK and SW) using the DNeasy Blood and Tissue Kit (Qiagen, Hilden, Germany), as per the manufacturer’s protocol.

PCR amplification targeting seven conserved housekeeping genes (*aro*C, *dna*N, *hem*D, *his*D, *pur*E, *suc*A, and *thr*A) was performed as previously described by Bell *et al*. [18] and Kidgell *et al*. [19]. Primer sequences and annealing temperatures are listed in Table 2 and were referenced from the EnteroBase database (https://enterobase.readthedocs.io/en/latest/mlst/mlst-legacy-info-senterica.html) [20]. Amplified products were sequenced through the Sanger method, assembled, and analyzed using Geneious Prime 2023.2.1. Sequence type (ST) numbers were assigned by submitting assembled sequences to the EnteroBase database (https://enterobase.warwick.ac.uk/species/index/senterica) [21]. When a sequence matched an existing ST, the associated serovar was assigned accordingly.

### Phylogenetic analysis

Seventeen *Salmonella* isolates (Table 3) were subjected to 16S rRNA gene sequencing using published primers [22], as detailed in Table 2. The resulting sequences were compared with GenBank records using the BLAST tool hosted by the National Center for Biotechnology Information. Phylogenetic analyses were conducted using Geneious Prime 2023.2.1. The 16S rRNA and MLST sequences were globally aligned at 70% similarity. A neighbor-joining phylogenetic tree was constructed using the Tamura-Nei genetic distance model with 1,000 bootstrap replicates to assess tree reliability. No outgroup was designated. Phylogenetic trees were edited and visualized using FigTree software v1.3.1 (FigTree, Edinburgh, UK).

**Table 3:** Phenotypic and genotypic characterization of *Salmonella* isolates recovered in Chonburi, Thailand, 2021–2023.

Isolate	Source	Location	Serovar		Sequence type (ST)^c^	Antimicrobial resistance patterns (R-patterns)

			Kauffman-White	7-gene MLST^c^		
S09	Macaque feces	Bang Phra, Chonburi	Uganda	Uganda	684	AMP AZI CHL COL SMX TET TGC TMP*
S10	Macaque feces	Bang Phra, Chonburi	Corvallis	Albany	292	AMP AZI CIP COL GEN NAL SMX TET TGC*
S11	Feed	Bang Phra, Chonburi	Weltevreden	Uganda	684	AMP CIP COL NAL TET TGC*
S13	Macaque feces	Bang Phra, Chonburi	Weltevreden	Weltevreden	365	Pan-susceptible
S14	Soil	Bang Phra, Chonburi	Hvittingfoss	Hvittingfoss	446	Pan-susceptible
S15	Dog feces	Bang Phra, Chonburi	Cerro	Cerro	1593	AMP AZI CIP SMX TET TGC*
S16	Macaque feces	Bang Phra, Chonburi	Corvallis	Corvallis	1541	AMP FOT TAZ CHL GEN SMX TET TGC TMP*
S21	Macaque feces	Bang Phra, Chonburi	Corvallis	Corvallis	1541	AMP CHL COL GEN TET TGC*
S22	Macaque rectal swab	Bang Phra, Chonburi	Rissen	Rissen	469	AMP TET TGC
S12^a^	Drain water	Goat farm, Chonburi^b^	Virchow	Virchow	359	n/a
S20^a^	Soil	Swine farm, Chonburi^b^	Stanley	Typhimurium	34	n/a
S23^a^	Turtle feces	Bang Phra, Chonburi	Rissen	Rissen	469	n/a
S24^a^	Pork	Local market, Chonburi	Kentucky	not defined	not defined	n/a
S25^a^	Pork	Local market, Chonburi	Agona	not defined	not defined	n/a
S26^a^	Pork	Local market, Chonburi	Rissen	not defined	not defined	n/a
SK [14]	Chicken feces	Poultry farms, Chonburi	Kentucky	Kentucky	198	n/a
SW [15]	Environment	Poultry farms, Chonburi	Weltevreden	Weltevreden	321	n/a

a Isolates were not included in this study; n/a: not applicable

b Demonstrated livestock farms, Faculty of Agriculture and Natural Resources, Rajamangala University of Technology Tawan-ok, Bangphra, Chonburi

c Enterobase database (https://enterobase.warwick.ac.uk/species/index/senterica)

* Multidrug resistance (MDR)

AMP=Ampicillin, AZI=Azithromycin, CHL=Chloramphenicol, CIP=Ciprofloxacin, COL=Colistin, FOT=Cefotaxime, GEN = Gentamicin, NAL=Nalidixic acid, SMX=Sulfamethoxazole, TAZ=Ceftazidime, TET=Tetracycline, TGC=Tigecycline, TMP=Trimethoprim

## RESULTS

### *Salmonella* prevalence

The overall prevalence of *Salmonella* isolates from human–macaque interface locations in the Si Racha and Sattahip communities, Chonburi Province, Thailand, was 2.88% (9/313). In the Si Racha district, the prevalence was 4.92% (9/183), whereas no positive isolates were detected in the Sattahip district (Table 1). The prevalence of *Salmonella* in environmental samples (8.89%; 8/89) was significantly higher than that in macaque rectal swabs (0.45%; 1/224), with an odds ratio (OR) of 22 (95% CI: 2.71–178.84; p = 0.0002). Among the 70 pooled macaque fecal samples collected, five (7.14%) tested positive for *Salmonella*. In addition, one out of three pooled stray dog fecal samples (33.33%) collected near the temple community was positive (Table 1). *Salmonella* was also detected in one of four feed samples (25%) and one of ten soil samples (10%) collected from the Bang Phra area (Table 1). All drain water samples collected from the Navy Base Camp in Sattahip tested negative for *Salmonella*.

### *Salmonella* serovar distribution

The nine *Salmonella* isolates identified in this study were distributed among six different serovars (Table 3), as determined using the standard Kauffman–White classification scheme. Five isolates originated from pooled macaque feces and included serovars Corvallis (n = 3), Uganda (n = 1), and Weltevreden (n = 1). An additional *S*. Weltevreden isolate was recovered from macaque feed collected from baited cages. One isolate of *S*. Cerro was identified in pooled stray dog feces from the temple community. A single Hvittingfoss serovar was detected in soil samples from the same area. One distinct isolate of the Rissen serovar was recovered from a macaque rectal swab. Minimal overlap in serovar distribution was noted among sample sources, except for serovar Weltevreden, which was found in both macaque feces and feed. Discrepancies were observed between traditional serotyping and the seven-gene MLST classification. For example, isolate S10 was initially identified as serovar Corvallis but was later reclassified as serovar Albany, while isolate S11, originally classified as serovar Weltevreden, was reidentified as serovar Uganda using MLST. Furthermore, sequence type (ST) numbers were assigned and correlated with serovar identities based on the sequencing of seven housekeeping genes (Table 3).

### Antimicrobial resistance profiles

All nine *Salmonella* isolates (one macaque-derived and eight environmental) were subjected to AST using the Sensititre® EUVSEC panel, which included 14 antimicrobial agents. A squashtogram was generated to illustrate the MIC distributions (Table 4), and the AMR profiles of the isolates are summarized in Table 3. The highest frequency of resistance was observed against tetracycline and tigecycline, with 77.78% (7/9) of isolates affected. This was followed by resistance to ampicillin, detected in 66.67% (6/9) of isolates (Table 4). Colistin resistance was identified in three isolates from macaque feces and one from a feed sample. None of the isolates exhibited resistance to meropenem. No identical resistance patterns (R-patterns) were observed among the AMR isolates. Moreover, most AMR isolates (6/7; 85.71%) – originating from macaque feces, stray dog feces, and feed – were classified as MDR, exhibiting resistance to at least three antimicrobial classes. Only the isolate recovered from the rectum of a macaque showed resistance to fewer than three antimicrobial classes, specifically ampicillin and the tetracycline group. In addition, two isolates – one from soil and one from pooled macaque feces – were pan-susceptible.

**Table 4:** Resistance and MIC distribution of *Salmonella* isolated from macaques and the environment, Chonburi, Thailand (n = 9).

^a^AM	^b^R (%)	^c^Distribution of MICs in μg/mL (%)														

		0.015	0.03	0.06	0.25	0.5	1	2	4	8	16	32	64	128	256	>1024
AMP	6 (66.67)						11.11		11.11	11.11		**11.11**		**55.56**		
AZI	4 (44.44)									33.33	22.22	**11.11**	**11.11**	**22.22**		
CHL	3 (33.33)									55.56	11.11				**33.33**	
CIP	3 (33.33)		44.44		11.11	11.11	**11.11**		**22.22**							
COL	4 (44.44)						55.56					**44.44**				
FOT	1 (11.11)				44.44	44.44				**11.11**						
GEN	2 (22.22)					33.33	11.11	22.22					**33.33**			
MEM	0		77.78	22.22												
NAL	2 (22.22)								44.44	33.33					**22.22**	
SMX	4 (44.44)									11.11	11.11	33.33				**44.44**
TAZ	1 (11.11)					77.78	11.11				**11.11**					
TET	7 (77.78)							22.22					**55.55**	**22.22**		
TGC	7 (77.78)					22.22	**22.22**	**33.33**	**22.22**							
TMP	2 (22.22)				22.22		55.56						**22.22**			

a Antimicrobials and their respective ranges of dilutions tested with breakpoints (μg/mL): AMP=Ampicillin (1–64; ≥32), AZI=Azithromycin (2–64; ≥32), CHL=Chloramphenicol (8–128; ≥32), CIP=Ciprofloxacin (0.015–8; ≥1), COL=Colistin (1–16; ≥4), FOT=Cefotaxime (0.25–4; ≥4), GEN = Gentamicin (0.5–32; ≥16), MEM=meropenem (0.03–16; ≥4), NAL=Nalidixic acid (4–128; ≥32), SMX=Sulfamethoxazole (8–1,024; ≥512), TAZ=Ceftazidime (0.5–8; ≥16), TET=Tetracycline (2–64; ≥16), TGC=Tigecycline (0.25–8; >0.5), TMP=Trimethoprim (0.25–32; ≥16), MIC=Minimum inhibitory concentration

b Number and percentage of resistant isolates to each antimicrobial agent

c The bold numbers indicate the percentages that exceed the resistance breakpoints

### Phylogenetic analysis

**Figure 3:**
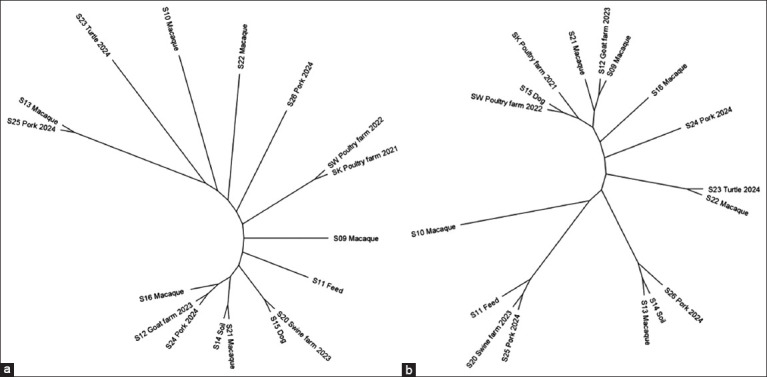
Phylogenetic diversity of *Salmonella* isolates based on 16S rRNA gene sequences (a) and 7-housekeeping gene sequences (b). The isolates were recovered from macaques, their shared environment, and other comparative sources in Chonburi, Thailand. The isolate labels correspond to the data presented in Table 3. Phylogenetic analyses were performed using Geneious Prime.

The phylogenetic tree based on 16S rRNA gene sequences (Figure 3a) for 17 isolates – comprising nine from this study and eight comparative isolates from Chonburi – did not reveal distinct clustering patterns according to sample source or serovar. Isolates re- covered from macaque feces (e.g., S13, S16, S21), environmental sources (e.g., S12, S14, S15, S20), and pork samples (e.g., S24, S25) clustered in close proximity. Notably, isolates from pooled macaque feces were dispersed throughout the phylogenetic tree, suggesting possible environmental mixing from multiple sources.

Similarly, the phylogenetic tree constructed using sequences of seven housekeeping genes (Figure 3b) revealed small, overlapping clusters comprising isolates from varied origins. These included the livestock farm environment (e.g., S12, S20, SK, SW), residential areas and pork (e.g., S14, S15, S25, S26), and macaques and their surrounding environments (e.g., S9, S11, S12, S13, S21). Some isolates obtained at different time points clustered together, indicating persistence and temporal continuity of certain STs. No clear clustering based on *Salmonella* serovar was observed, though the Rissen serovar, recovered from macaque rectal swabs and turtles, formed a distinct group. In addition, isolates from pooled macaque feces were widely distributed across the phylogenetic tree, further supporting evidence of environmental contamination within shared habitats.

## DISCUSSION

This study aimed to elucidate the prevalence, AMR profiles, and molecular characteristics of *Salmonella* isolates from free-ranging long-tailed macaques and their surrounding environments in Chonburi Province, Thailand, under a One Health framework.

### Prevalence and spatial patterns of *Salmonella*

This study elucidated the prevalence and distribution of *Salmonella* and its AMR profiles isolated from macaques and environmental sources in Chonburi, where free-roaming long-tailed macaques coexist and interact with human communities. The overall prevalence of *Salmonella* in the study population was 2.88%. Notably, *Salmonella* was detected exclusively in the Si Racha district (4.92%), with no positive cases identified in Sattahip. This disparity may be explained by the greater urbanization of Si Racha, which is characterized by close proximity between residential areas, livestock production, and wildlife habitats. Free-roaming long-tailed macaques are commonly seen throughout the study area, as shown in Figure 1. In contrast, the macaques in Sattahip primarily roam the natural habitats surrounding the navy camp, with limited interaction with residential zones. Furthermore, the community in Sattahip is quieter and more isolated, with a predominantly military population.

Compared with other locations in Thailand, the prevalence of *Salmonella* isolated from fecal specimens of free-living long-tailed macaques in Lopburi Old Town was 1.71% [4]. Another study on the prevalence of the Enterobacteriaceae family in wild long-tailed macaques reported a 10% isolation rate of *Salmonella* spp. across central Thailand, including the Phichit, Uthai Thani, and Suphan Buri provinces [7]. Consistent with findings from other regions, the prevalence of *Salmonella* in free-ranging macaques and non-human primates ranges from 2.2% to 13.9% [3, 5, 23–25]. In contrast, our study recorded a lower prevalence of 0.45% in macaque rectal samples and a higher prevalence of 8.89% in environmental samples. The occurrence of *Salmonella* in on-ground macaque feces and the surrounding environment was significantly higher than that in rectal samples (OR = 22; 95% CI: 2.71–178.84; p = 0.0002). Although studies by Rahman *et al*. [3], Boonkusol *et al*. [4], and Balasubramaniam *et al*. [7] have reported that free-roaming macaques serve as reservoirs for various zoonotic pathogens, definitive transmission routes remain unclear, as these studies primarily relied on fecal examinations rather than rectal swabs. Our findings suggest the possibility of reverse zoonotic *Salmonella* transmission between humans, macaques, and shared environmental reservoirs. Nevertheless, the moisture content in macaques’ rectal cavities may influence the viability of bacterial cultures and the transport of swabs, potentially affecting isolation rates. Although all macaques in this study passed basic health screenings, these potential sampling biases should be considered in future research.

### AMR Patterns and MDR

The *Salmonella* isolates identified in this study exhibited high levels of resistance across multiple antimicrobial classes, with a particularly high incidence of MDR. The most frequently observed resistance (77.78%) was to tetracycline and tigecycline, which belong to the same class. This finding is consistent with a previous report from Bangladesh, where rhesus macaques living in close contact with humans demonstrated the highest resistance levels to these drugs [3]. High rates of resistance to ampicillin, azithromycin, and sulfamethoxazole were also detected. These results are in agreement with several global studies on AMR in livestock, food products, wildlife, and environmental isolates [8, 26–30].

Our study identified colistin-resistant *Salmonella* strains in the feces of macaques. Colistin, which is increasingly employed in livestock production across Asia [31], has attracted considerable global attention due to its rising resistance profile [32]. Although the presence of colistin-resistant *Salmonella* in wildlife has not been widely reported, our findings demonstrate its emergence within macaque populations in this locality.

### Potential for environmental dissemination and reverse zoonosis

We also observed diverse resistance patterns and MDR profiles in the majority of *Salmonella* isolates, particularly those derived from pooled macaque feces, pooled dog feces, and feed samples. This suggests that free-ranging macaques and their surrounding environment may serve as amplifiers and disseminators of AMR *Salmonella*. Interestingly, one AMR isolate directly recovered from a macaque rectum exhibited resistance to only two antimicrobial classes. This observation raises concern about the potential reverse transmission of AMR bacteria, particularly in areas where macaques reside in close proximity to human populations. As is widely known, free-ranging macaques in Thailand do not receive antimicrobial treatments. Thus, AMR transmission likely occurs through direct or indirect contact with humans, domestic animals, or contaminated environmental reservoirs in shared habitats.

### Serovar distribution and public health relevance

In this study, six distinct *Salmonella* serovars were identified, with no clear associations between serovar distribution, sample source, or resistance pattern. While some studies by Pavon *et al*. [33] and Borges *et al*. [34] suggest that specific serovars influence the distribution of AMR, virulence, or plasmid carriage, our findings do not support such associations. The predominant serovar detected was *S*. Corvallis, isolated from the feces of macaques. This serovar has been increasingly reported in human infections, livestock, and food products across Asia, raising public health concerns regarding its potential for antibiotic resistance [35–38]. To our knowledge, this is the first report of MDR *S*. Corvallis in macaques in Thailand.

*S*. Weltevreden, a known diarrheagenic pathogen in humans [39, 40], was another frequently isolated serovar in this study. The prevalence of *S*. Weltevreden has risen substantially across a wide host range, including humans, farm animals, aquaculture species, reptiles, and produce, particularly in Southeast Asia [39–43]. The distinct serovar *S*. Cerro, identified in stray dog feces and resistant to six antimicrobials, is rarely reported in Southeast Asia but has been frequently documented in U.S. dairy herds [44, 45]. The presence of this serovar in a new host and geographical context necessitates continued surveillance.

### Molecular typing and serotyping approaches

MLST, which analyzes the sequences of conserved housekeeping genes, was used for serotyping and ST determination of the *Salmonella* isolates. Chattaway *et al*. [46], Luo *et al*. [47], and Yan *et al*. [48] have advocated for reporting *Salmonella enterica* subtypes based on ST followed by serovar assignments. Most of our MLST-based identifications were consistent with the Kauffman–White classification, except for isolates S10 (*S*. Corvallis) and S11 (*S*. Weltevreden), which were reclassified as *S*. Albany and *S*. Uganda, respectively. We recommend applying both methods in parallel for serotyping confirmation or adopting advanced genomic tools such as core genome MLST (cgMLST), which targets 3,002 conserved loci, or whole-genome MLST (wgMLST), which includes all pan-genomic coding sequences [48, 49].

### Phylogenetic inference and epidemiological interpretation

Phylogenetic analyses were conducted to examine epidemiological relationships among *Salmonella* isolates from macaques and environmental sources. The 16S rRNA gene-based tree revealed no clear clustering by serovar or sample source, indicating a random distribution of the isolates. Similarly, the MLST-based phylogeny showed modest clustering among isolates from different sources, but definitive groupings were not observed. Some isolates obtained from different time periods clustered together, suggesting temporal continuity of circulating strains. Isolates from pooled macaque feces were dispersed throughout both trees, implying environmental cross-contamination or shared microbial reservoirs.

In contrast to earlier studies demonstrating clear clustering by source or serovar [48, 49], our findings lacked such patterns. While 16S rRNA sequencing remains an accessible and widely used method for classification, phylogenetic assessment, and outbreak investigation [49–51], its resolution is limited in distinguishing closely related strains [49, 50]. To enhance phylogenetic accuracy, we recommend incorporating additional targets such as *avr*A and *spv*C genes [50] or utilizing 7-gene MLST. Nevertheless, MLST did not provide sufficient discriminatory power to infer precise transmission relationships in this study. To our knowledge, this represents the first molecular characterization of AMR *Salmonella* from macaques and their environment in Thailand under a One Health perspective.

## CONCLUSION

This study presents the first molecular characterization of AMR *Salmonella* at the human–macaque–environment interface in Thailand, providing critical insights into the role of free-ranging long-tailed macaques and shared environments in the ecology of AMR. The overall prevalence of *Salmonella* was 2.88%, with all isolates originating from the urbanized Si Racha district. Significantly higher detection rates were observed in environmental samples (8.89%) compared to macaque rectal swabs (0.45%) (OR = 22; 95% CI: 2.71–178.84; p = 0.0002), suggesting that environmental reservoirs may play a key role in the transmission cycle.

Among the nine confirmed isolates, 77.78% were resistant to tetracycline and tigecycline, while 66.67% exhibited ampicillin resistance. MDR was observed in 85.71% of the AMR strains, most notably among samples from pooled macaque feces, stray dog feces, and feed sources. The emergence of colistin-resistant *Salmonella* in macaques – an antibiotic of last resort – further raises public health concerns. Six distinct serovars were identified, including *S*. Corvallis and *S*. Weltevreden, which are increasingly associated with human and foodborne infections across Asia.

Practical applications of these findings underscore the urgent need for integrated One Health surveillance that encompasses all three domains: wildlife, environmental, and human. Public health authorities should consider implementing interventions such as improved waste management, limiting wildlife access to anthropogenic food sources, and enhancing biosecurity measures around macaque habitats and tourist areas.

The strengths of this study lie in its multi-disciplinary approach, which incorporates traditional micro-biological techniques with molecular methods (MLST and 16S rRNA sequencing) to assess resistance profiles, serovar diversity, and phylogenetic relationships within a wildlife–environment continuum.

However, several limitations should be acknowledged. The relatively small number of *Salmonella*-positive samples limited the genetic diversity observed and restricted the ability to draw serovar-specific or source-specific conclusions. Sample size variability was influenced by uncontrollable factors, such as troop leader behavior and environmental conditions that affected macaque capture. Furthermore, reliance on 1–7 genes for phylogenetic analysis may not provide sufficient resolution to trace transmission events or detect subtle genetic differences. Swab-based sampling may also have been affected by rectal moisture, which could have potentially influenced bacterial recovery.

Future studies should incorporate whole-genome sequencing approaches (e.g., cgMLST, wgMLST, wgSNP) to achieve higher resolution in tracking transmission pathways, resistance gene dynamics, and interspecies transmission. Expanding geographic scope and sampling frequency, along with the inclusion of human and domestic animal samples, will be essential for comprehensive risk assessment and control strategies.

In conclusion, this study highlights the ecological complexity and public health risks posed by antimicrobial-resistant *Salmonella* in urban macaque habitats. The findings call for proactive, cross-sectoral interventions under the One Health framework to mitigate zoonotic and environmental transmission risks in rapidly developing regions.

## DATA AVAILABILITY

All the generated data are included in the manuscript.

## AUTHORS’ CONTRIBUTIONS

SP: Designed the study, performed data analysis, MLST and phylogenetic analysis, and drafted and revised the manuscript. DT and SW: Designed the study, collected the samples, and drafted and revised the manuscript. PP: Collected the samples and contacted local officers. KN: Performed bacterial isolation and drafted the manuscript. SM: Performed bacterial isolation, AST, PCR, and molecular detection. All authors have read and approved the final manuscript.
